# Chemical Characteristics and Oxidative Stability of Buffalo Mozzarella Cheese Produced with Fresh and Frozen Curd

**DOI:** 10.3390/molecules26051405

**Published:** 2021-03-05

**Authors:** Simona Rinaldi, Giuliano Palocci, Sabrina Di Giovanni, Miriam Iacurto, Carmela Tripaldi

**Affiliations:** Consiglio per la Ricerca in Agricoltura e L’analisi Dell’economia Agraria (CREA), Centro di Ricerca Zootecnia e Acquacoltura, Via Salaria, 31, 00015 Monterotondo (RM), Italy; giuliano.palocci@crea.gov.it (G.P.); sabrina.digiovanni@crea.gov.it (S.D.G.); miriam.iacurto@crea.gov.it (M.I.); carmela.tripaldi@crea.gov.it (C.T.)

**Keywords:** antioxidant activity, lipid oxidation, protein oxidation, MDA, carbonyls

## Abstract

Milk and dairy products can have variable contents of antioxidant compounds that contribute to counteract the oxidation of lipids and proteins during processing and storage. The content of active antioxidant compounds is closely linked to their protection by oxidation. Freezing is one of the factors that can reduce antioxidant activity. Freezing of milk or curd is frequently used in case of the seasonality of milk production and/or seasonal increased demand for some products. In this paper, the effect of using frozen curd on the oxidative stability of buffalo Mozzarella cheese was evaluated. Samples of buffalo Mozzarella with different frozen curd content (0%, 5%, 20%, and 50%) were produced and analyzed at one and nine days. Mozzarella cheese with higher frozen curd content had a significant increase in redox potential parallel to the decrease in antioxidant activity, showing less protection from oxidation. Lipid and protein oxidation, expressed respectively by malondialdehyde and carbonyl content, increased significantly with increasing frozen curd. At nine days, carbonyls significantly increased while malondialdehyde content did not vary, showing that during storage, fat was more protected from oxidation than protein. The average carbonyl levels were comparable to those of some cooked cheeses, and the malondialdehyde levels were even lower. The results of this study stimulate the investigation of new strategies to decrease the oxidative damage in cheeses produced in the presence of factors decreasing oxidative stability.

## 1. Introduction

Buffalo Mozzarella is a fresh Italian *pasta filata* cheese whose excellent characteristics are known to many consumers. In 1996 the European Union recognized the *Mozzarella di Bufala Campana* as a Protected Designation of Origin (PDO) cheese (EC Regulation No. 1107/96) [1]. Most of the production of buffalo Mozzarella cheese is concentrated in the area of origin.

According to data over the last years, two-thirds of the total production of buffalo Mozzarella cheese had PDO status [2]. The total production of both PDO and non-PDO buffalo Mozzarella cheese is increasing. The production of *Mozzarella di Bufala Campana* PDO ranged from 41,295 tons in 2015 to 50,176 tons in 2019 [2].

Cheese-making steps and storage conditions could affect the characteristics and oxidative stability of cheese, influencing the formation of oxidation products. This has also been observed in buffalo Mozzarella cheese [3,4].

Dairy products are subjected to lipid and protein oxidation during production processes, and the content of compounds with antioxidant activity is closely linked to their protection by oxidation [3,5]. Milk and dairy products have variable content of antioxidant compounds such as enzymes, vitamins, and peptides [6,7]. In particular, the presence and concentration of antioxidants such as vitamins E and A and β-carotene strongly depend on feeding management [8,9]. The intake of vitamins and antioxidants within the diet is transferred to milk. Supplementing vitamin E in the diet can increase the antioxidant capacity of milk and decrease the products of oxidation [10]. In buffalo milk, there are not carotenoids but a high content of vitamin A. However, its total vitamin A per unit weight of fat is lower than in cow milk due to the higher fat content in buffalo milk [11,12].

The oxidative stability of milk and dairy products during production and storage has great importance for human nutrition, also contributing to body defenses against oxidation [13]. The lipid oxidation of dairy products strongly contributes to the degradation of the nutritional and sensory properties, while antioxidant molecules could play a protective role of lipids from oxidative stress during manufacture and storage [3,14]. The terminal phase of lipid oxidation leads to the formation of different products: aldehydes, ketones, hydrocarbons, alcohols, and acids [15]. The content of malondialdehyde (MDA), a highly reactive aldehyde derived by lipid oxidation, is often used as a marker of oxidative damage in cheese [3,16].

In dairy products, even protein oxidation concurs to oxidative damage mostly induced by heat treatment during the cheese-making process [3,4] or by light exposure during storage [17]. The amino acid residues most sensitive to oxidation are aromatic amino acids such as tryptophan and tyrosine, but aliphatic residues may also be oxidized [18]. The levels of protein-bound carbonyls can represent an index of global protein oxidation irrespective of the initiating radical species and chemical process [19,20]. Carbonyl groups derive from protein oxidation not only by the direct oxidation of amino acids by free radicals but also by the secondary oxidation of proteins during lipoperoxidation and glycation. In particular, glycation or the Maillard reaction occurs between amino groups (predominantly lysine and arginine groups) and carbonyl groups of reducing sugars during cheese manufacture and storage, resulting in the production of dicarbonyl intermediates [4]. Reactive dicarbonyl compounds or α-β-unsaturated aldehydes originating from the Maillard reaction or from lipid oxidation can lead to the formation of protein-bound carbonyls [19,21].

It has long been known that milk and its derivatives are frozen to preserve them or extend shelf life. Freezing is frequently used in case of the seasonality of milk production and/or seasonal increased demand for some products [22]. In Italy, the market demand for buffalo Mozzarella increases a lot during the hot season. The use of frozen milk or curd makes it possible to satisfy the increased consumption of this product at certain times of the year. The PDO specifications of *Mozzarella d Bufala Campana* require that the milk used for production must be processed within 60 h of milking; therefore, frozen milk or curd cannot be used in dairies that adhere to the PDO mark.

In general, freezing is considered a good method to preserve food for long periods and to use the frozen product when it is needed, but physical and chemical modifications occur during freezing, storage, and thawing due to alterations in composition and microstructure [23]. During milk freezing, ice crystals formation could cause fat coalescence due to the breakdown of fat globule membranes, and consequently, the fat fraction loses its emulsified state [24,25].

Recently, a study on buffalo Mozzarella cheese produced with the addition of frozen curd was carried out, with the final purpose to evaluate the possibility to trace frozen milk or curd in buffalo Mozzarella PDO cheese [26,27], but there are no studies focused on oxidative modifications due to frozen curd addition. In fact, the freezing can induce chemical modifications as lipid and protein oxidation or degradation of antioxidant molecules [28]. Chemical modifications and oxidative damage could occur during the storage of frozen curd. The increase in oxidation degree of frozen curd could induce chemical modifications in Mozzarella cheese produced with frozen curd compared to that produced with fresh curd alone.

In this study, we evaluated chemical characteristics and oxidative modifications of buffalo Mozzarella cheese produced with fresh and frozen curd. The objective is to verify the modifications due to the use of frozen curd in buffalo Mozzarella cheese-making and to check oxidation stability during storage.

## 2. Results and Discussion

### 2.1. Chemical Characteristics

The chemical characteristics of fresh and frozen curd used for buffalo Mozzarella cheese-making in this study are reported in Table 1. These are the results of analyses carried out on fresh and frozen curd used in the three replicates. The determination of the characteristics of the curd was carried out in order to better evaluate the chemical modifications induced by the freezing of the curd on the Mozzarella samples. The results show that freezing led to statistically significant modifications in some parameters of curd (Table 1) as discussed below in the evaluation of Mozzarella cheese characteristics.

Chemical characteristics of Mozzarella cheese produced by adding frozen curd (FC) at various percentages (0%, 5%, 20%, and 50%) are reported in Table 2. The analyses were carried out at one (T1) and nine days (T9) from cheese samples produced in three replicates (4 × 2 × 3).

The pH value of Mozzarella cheese was between 5.39 and 5.42, showing a steady trend without significant variations according to FC%. Instead, the pH increased from 5.34 to 5.44 (*p* < 0.0001) during nine days of storage.

The moisture % in Mozzarella cheese decreased significantly (*p* < 0.05) with the addition of 20% FC compared to 0% and 5% FC, while for the ash content, there was a tendency to increase. Both the moisture % (*p* < 0.0001) and ashes % (*p* < 0.05) were higher in Mozzarella cheese at T9 compared to T1.

The protein content increased significantly (*p* < 0.0001) while the fat content and fat/protein ratio decreased significantly (*p* < 0.01; *p* < 0.0001) with the increase in FC%. Significant (*p* < 0.0001) decrease in both protein and fat content was found in Mozzarella cheese after nine days of storage compared to fresh cheese, while the fat/protein ratio was invariant. For all parameters, no interaction was found between the two factors (FC% × Storage).

The chemical characteristics of the Mozzarella samples showed that the higher the FC content, the lower the fat content and the fat/protein ratio. However, the minimum threshold of fat/dry matter content of 52% set by the *Mozzarella di Bufala Campana* PDO specification was largely respected. These values in our samples ranged from 63% with 0% FC to 58% with 50% FC. More factors may have determined the lower fat content of samples with 50% FC. One of these factors could be the lower fat content in frozen curd compared to that in the fresh curd (29.10% vs. 30.07%, *p* < 0.05, Table 1).

The effect of the moisture content of the curd on that of Mozzarella is more difficult to explain, considering that this content was lower in fresh curd (43.4%) and higher in frozen one (46.6%, *p* < 0.005, Table 1). Other factors that may have favored lower fat recovery and lower moisture content in Mozzarella cheese with a higher percentage of frozen curd are the conditions of freezing and thawing, and stretching of the curd.

There is little data on the effects of using frozen curd in the production of buffalo Mozzarella cheese. According to Manzo [26], the addition of higher FC levels to the fresh curd had a depressing effect on the moisture and fat content of buffalo Mozzarella cheese. In samples containing 40% FC, the fat/protein ratio reported by Manzo [26] was 0.96, while it was 1.39 in our 50% FC samples. The fat/dry matter ratio of Mozzarella cheese with 40% FC reported by Manzo [26] was also very low (47.3%), far below the minimum threshold of 52%. This ratio was largely influenced by the very low moisture content in Mozzarella samples.

Addeo et al. [29] found that the fat content of buffalo Mozzarella cheese produced with frozen milk was affected by its freezing conditions and, in particular, by thawing conditions. According to these authors, part of the fat was separated during the milk thawing phase, and this fat was not incorporated into the curd. However, when Mozzarella cheese was made with milk that was rapidly frozen and thawed, its fat content was similar to that of Mozzarella cheese made with fresh milk [29]. At the moment, there is no evidence that further improving the freezing and thawing conditions of the curd have positive effects on the fat content of Mozzarella cheese. According to Alichanidis [30], the lower moisture content in cheese made from frozen curd may be due to some changes in the structure of the casein micelles, which result in a reduced ability of the curd to retain whey.

The increasing protein content in Mozzarella cheese when FC increased could be directly related to a reduction in fat content. Buffalo Mozzarella cheese has a structure formed by a casein network that delimits the alveoli in which water and fat globules are contained. In such a structure, a reduction in the fat content leads to a proportional increase in the protein content [29].

Our buffalo Mozzarella samples were produced with static freezing of the curd, but the characteristics of the freezing cell allowed a reduction in freezing time to 3–4 h. The time spent defrosting the curd was the same. Therefore, the results obtained have to be interpreted in the context of these curd freezing and thawing conditions. Other trials are planned to evaluate the effect of the different freezing and thawing methods on the Mozzarella cheese composition. It should be noted that the moisture and fat content of Mozzarella affects the cheese yield. Citro [31] argues that the stretched cheese yield varies according to the composition, processing type, and moisture of the final product.

During the storage of buffalo Mozzarella cheese for a period of nine days, all the characteristics changed significantly. The pH value increased as well as the moisture and ash content. The protein and fat content and the fat/protein ratio decreased. Our samples of buffalo Mozzarella cheese were packaged and stored at 4 °C in a preserving liquid containing tap water, citric acid, and salt. The storage in preserving liquid prevents the formation of rind and conserves the soft-springy texture in the high moisture Mozzarella cheese. However, this kind of storage also causes the exchange of water and soluble components between Mozzarella cheese and preserving liquid, and this transfer could explain the increase in moisture and ash content during storage [32,33]. In particular, the higher ash content was due to the increased salt content in the stored Mozzarella cheese (unpublished data). The decrease in protein and fat could be the effect of the dilution of these components due to the increased moisture content of the Mozzarella samples. The increase in pH could indicate a reduced concentration of lactic acid bacteria, which produce lactic acid and contribute to lowering the pH [34].

### 2.2. Oxidative Characteristics

Oxidative characteristics of fresh and frozen curd used in buffalo Mozzarella cheese-making are reported in Table 3.

The results show that statistically significant modifications occurred in most oxidative parameters of frozen curd compared to those of fresh one, as discussed below, together with the Mozzarella cheese oxidative characteristics.

Oxidative characteristics of buffalo Mozzarella cheese are shown in Table 4.

#### 2.2.1. Redox Potential Determination

The redox potential increased significantly (*p* < 0.0001) with increasing frozen curd percentage in Mozzarella samples (186 mV in 0% FC, 210 mV in 50% FC, Table 4). This trend could be due to the higher redox potential of frozen curd compared to fresh curd (226 mV vs. 163 mV, *p* < 0.0001, Table 3).

**Figure 1:**
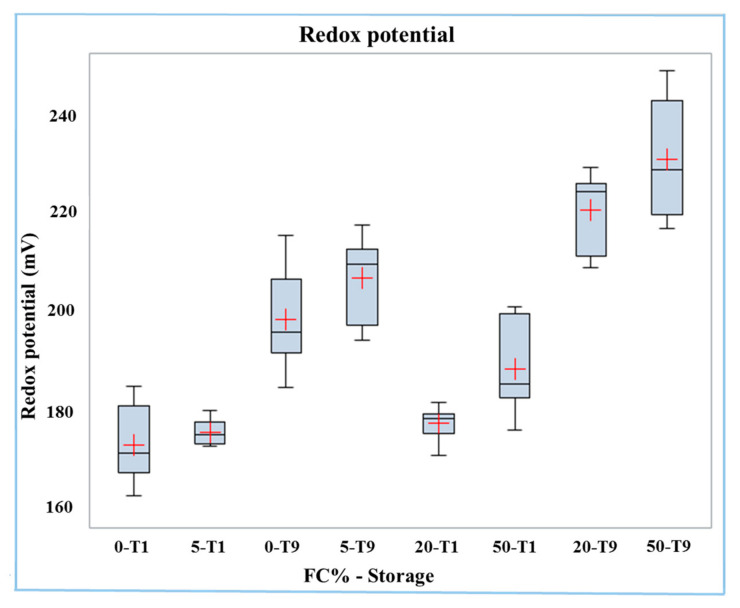
Boxplot of redox potential in buffalo Mozzarella cheese produced with frozen curd (FC) preserved for one (T1) and nine days (T9).

The redox potential was on average higher (*p* < 0.0001) in Mozzarella cheese samples after nine days of storage (T9) than in those of fresh Mozzarella cheese (T1) (215 mV vs. 179 mV). The interaction of the two factors was significant (*p* < 0.05), and the redox potential data is reported in the boxplot in Figure 1. The effect of the frozen curd content on increasing the redox potential was most relevant in stored samples. In effect, the samples with the highest redox potential were those with 20% and 50% frozen curd stored for nine days, while the samples having the lowest redox potential were those with 0% and 5% of frozen curd at T1.

The redox potential was higher than that previously observed in buffalo Ricotta cheese (121–134 mV) [35] and similar to the average level in industrial cow Ricotta cheese (183 mV) [36].

The redox potential determination in milk and cheese could be a useful electrochemical method to test the susceptibility to oxidation, but its routine measurement has limitations due to the separation between hydrophobic and hydrophilic phases [37,38]. Abraham et al. [39] reported that redox potential was heterogeneous in Camembert cheese due to a rind to core gradient where the surface was oxidizing (+330 to +360 mV) and the core was reducing during ripening (–300 to –360 mV). Negative values at the cheese core probably reflected the oxygen gradient decreasing from rind to core. Products with a positive redox potential have oxidant properties, and the levels are influenced particularly by their chemical compositions, heat treatments, exposure to atmospheric oxygen, and microbial metabolic processes [39].

#### 2.2.2. Antioxidant Activity Determination

There are several methods to evaluate the antioxidant activity in milk [40,41], and they are useful tools in estimating the total ability of the products to counteract the oxidation that normally occurs during processing and storage in milk and cheese. Milk is a complex system of pro and antioxidant components [6], and heat treatments can reduce its total antioxidant activity probably due to decreased activity of vitamins and enzymes.

The antioxidant activity of Mozzarella cheese (Table 4), determined as DPPH free-radical scavenging activity, decreased significatively (*p* < 0.0001) with the increase in the percentage of frozen curd in the samples, from 10.08 mmol Trolox eq/100 g (corresponding to 47.6%) in samples without frozen curd to 8.81 mmol Trolox eq/100 g (41.6%) in the samples with 50% FC. These values could depend on the higher antioxidant activity in fresh curd than in frozen curd (10.3 mmol Trolox eq/100 g vs. 9.4 mmol Trolox eq/100 g, *p* < 0.05, Table 3). No significant differences were found with respect to storage time of Mozzarella cheese (9.53–9.26 mmol Trolox eq/100 g corresponding to 45.0–43.7%).

In a previous study [35], the total antioxidant activity of Ricotta cheese increased slightly during storage from day one (13.69 mmol Trolox eq/100 g or 64.60%) to day 21 (14.05 mmol Trolox eq/100 g or 66.30%). Other studies reported that the antioxidant activity in Cheddar cheese increased during the first four months of ripening due to soluble peptides formation by proteolysis [42], and supplementation with selenium and vitamin E considerably improved the antioxidant capacity of fresh cheese, inhibiting the lipid oxidation during ripening [43]. Also, in Pecorino cheese supplemented with natural antioxidants, the antioxidant activity enhanced while the lipid oxidation decreased [44]. A similar increasing trend in antioxidant activity was also shown in other cheeses such as Gouda cheese fortified with mango kernel fat [45]. The decrease in antioxidant activity is mostly depending on the degradation of antioxidant components, such as vitamins and enzymes, while its increase in cheese could be related to the formation of water-soluble peptides during ripening which has antioxidant properties [42,46].

#### 2.2.3. Lipid Oxidation and MDA Content

The study of oxidation degree mainly uses methods that detect alterations in lipids and proteins, and the presence of oxidation products is considered a marker of oxidative damage [3,16]. As the final product of lipid oxidation, malondialdehyde (MDA) content was analyzed to test the presence of lipid oxidation in Mozzarella samples, and the results are presented in Table 4.

The MDA content increased significantly (*p* < 0.0001) with the increase in frozen curd, from 0.36 nmol/g in samples without frozen curd to 0.85 nmol/g in samples containing 50% FC. This increase in MDA levels may depend on the higher MDA content in frozen curd than in fresh one (1.22 nmol/g vs. 0.67 nmol/g, *p* < 0.0001, Table 3). Moreover, MDA content in Mozzarella samples remained essentially unchanged during the nine days of storage (0.55–0.59 nmol/g).

The average MDA content previously reported in Mozzarella cheese from bovine and buffalo milk (0.11–0.19 nmol/g [3]) was lower than the values found in our samples, including those produced only from fresh curd. The values reported in homogenized Ricotta cheese (1.51 nmol/g) were higher [35]. Values of semi-cooked and cooked cheeses varied from 1.28 nmol/g in Grana Padano cheese [3] to 1.80 nmol/g in Pecorino cheese [44]. The values of MDA content found in our buffalo Mozzarella cheese were lower than those found in other dairy products, although, according to some authors, the high fat content of buffalo Mozzarella cheese is a factor that predisposes it to lipid oxidation [13,17].

There are some reports in the literature on the effects of frozen storage on lipid oxidation in chicken and beef meat [47,48]. These findings indicate that lipid oxidation is accelerated with prolonged storage [49,50]. Although there is not any legislative limit to MDA concentration, Reitznerová et al. [51] reported MDA levels over 0.5 mg/kg (6.9 nmol/g) as an index of some oxidation and values above 1.0 mg/kg (13.9 nmol/g) as possibly unacceptable levels in meat.

#### 2.2.4. Protein Oxidation and Carbonyl Content

Carbonyl analysis is the most common method for determining protein oxidation of foods. Since carbonyls are the principal products of protein oxidation, the increase in total carbonyl groups is associated with an increase in protein oxidation [52].

**Figure 2:**
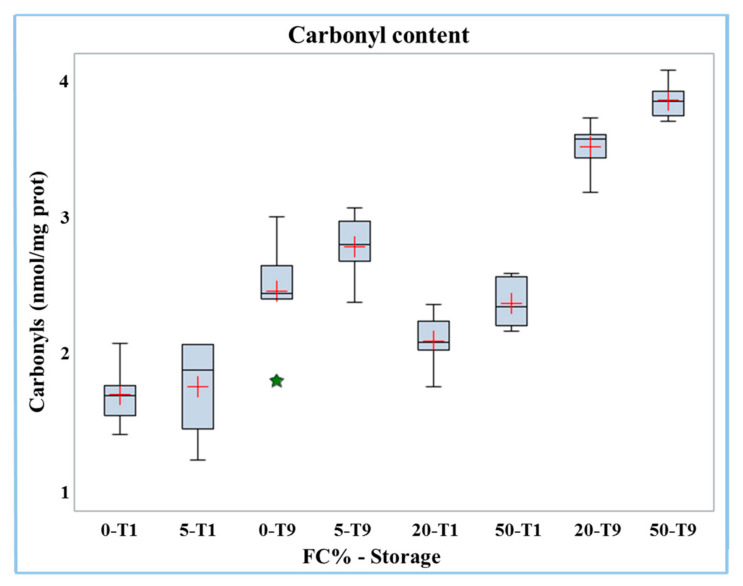
Boxplot of carbonyl content in buffalo Mozzarella cheese produced with frozen curd (FC) preserved for one (T1) and nine days (T9).

The carbonyl content increased significantly (*p* < 0.01) in frozen curd (2.13 nmol/mg protein) compared to fresh curd (1.54 nmol/mg protein, Table 3). The results of carbonyl analyses in Mozzarella cheese samples containing different percentages of frozen curd are presented in Table 4. Both the percentage of frozen curd and duration of storage had significant (*p* < 0.0001) positive effects on the carbonyls content in Mozzarella cheese. The interaction of the two factors (FC% and storage) was significant (*p* < 0.01); the carbonyl content data is reported in the boxplot in Figure 2.

The results showed that the increase in carbonyl groups with the percentage of frozen curd content became more evident in the samples after nine days of storage (at T1 from 1.72 to 2.39 nmol/mg protein while at T9 from 2.47 to 3.86 nmol/mg protein). Hence, the highest level of carbonyls was found in Mozzarella cheese containing 50% frozen curd after nine days of storage.

The values of carbonyls observed in our fresh samples were similar to those previously reported in buffalo Mozzarella cheese (1.95 nmol/mg protein) [4]. Higher and more variable carbonyl levels in cow Mozzarella cheese (on average 5.57 nmol/mg protein) are reported by the same authors. In particular, the carbonyl content after nine days of storage in 20% and 50% frozen curd samples was similar to that reported in Ricotta cheese (3.01–3.93 nmol/mg protein) [35] and in other cooked cheeses, such as Grana Padano, Caciocavallo, Provolone and Pecorino Romano (2.83–4.54 nmol/mg protein) [3]. The great variability of the carbonyl values found in different kinds of cheeses probably reflected the heterogeneity in heat treatments [4] and in the whole process.

Carbonyl content in proteins might be responsible for the formation of molecular cross-links, supporting the hypothesis that carbonyls might play a role in the formation of stable protein aggregates in processed foods. This role is also played by hydrophobic interactions, such as dityrosine and disulphide cross-linking, inducing structural modification and protein denaturation during heat treatment [4,21]. Some authors reported an increase in carbonyl groups during frozen storage of chicken meat and fish [47,53]. Therefore, the use of frozen curd increases protein oxidation, probably due to modification of amino acids, denaturation, loss of solubility, and related carbonyl increase, as reported in frozen meat [47,48,54]. There are many factors reported to have an influence on the occurrence and extent of protein oxidation in foods, including various processing factors such as aging, cooking, prolonged storage, freezing, and thawing [48,55]. The effect of time and storage conditions on protein oxidation may vary depending on the extent of previous treatments.

#### 2.2.5. Protein Denaturation and the FAST Index

The FAST index (Fluorescence of Advanced Maillard products and Soluble Tryptophan) has been used in previous works to estimate the changes induced in milk by heat-treatments [56,57]. In the present study, we determined the FAST index in cheese to assess the degree of protein denaturation by tryptophan content and Maillard’s Advanced Products (AMP) in Mozzarella cheese.

Tryptophan fluorescence (Trp-F) decreased significatively (*p* < 0.0001, Table 4) both with the increase in FC% in Mozzarella cheese and with storage time, but no interaction between the two factors was found. Trp-F decreased from 385 a.u. in 0% FC to 334 a.u. in 50% FC. There was also a reduction in Trp-F from 420 to 370 a.u. in frozen curd compared to the fresh one (*p* < 0.001, Table 3).

Tessier et al. [57] reported that the fluorescence of Trp in milk is positively related to the soluble protein content at pH 4.6 because the analysis is carried out on pH 4.6 soluble fraction of protein. Therefore, the decrease in Trp-F indicates both Trp decrease and the decrease in soluble proteins due to denaturation and precipitation of proteins. According to Soliman et al. [58], the soluble protein content could also decrease due to freezing storage.

Tryptophan is an essential amino acid that regulates numerous physiological functions, and its degradation during food processing decreases its nutritional supply [59,60].

AMP fluorescence (AMP-F) did not vary significantly with FC% increase in Mozzarella samples (range from 56.3 a.u. at 0% to 60.5 a.u. at 50%) while it decreased (*p* < 0.0001) from 62.5 a.u. at T1 to 54.5 a.u. at T9 (Table 4). No differences were found in AMP-F between fresh and frozen curd (Table 3). AMP content generally increased in samples subjected to high heat treatments, but there was no evidence of it increasing with freezing storage.

**Figure 3:**
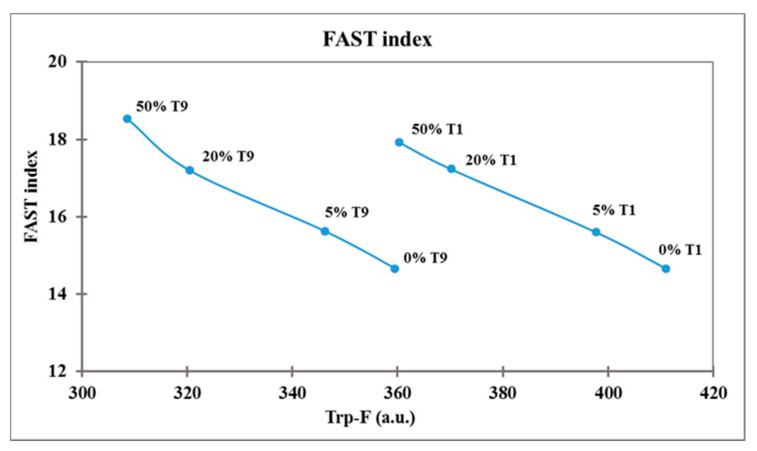
Relation between tryptophan fluorescence and FAST index in Mozzarella cheese samples.

The FAST index, calculated as AMP-F/Trp-F × 100, was higher in frozen curd than in fresh, depending on the decrease in Trp-F (Table 3). The FAST index significantly increased (*p* < 0.0001) with the increase in the frozen curd percentage in Mozzarella cheese (14.7 in 0% FC, 18.2 in 50% FC), due to the decrease in Trp-F, while it did not change with storage time (16.3–16.5), and the Trp-F/AMP-F rate remained unchanged (Table 4). The relation between Trp fluorescence and FAST index is reported in Figure 3, where each sample of Mozzarella cheese was plotted at T1 and T9 storage times.

The Trp-F varied as a function of the two factors tested, the percentage of frozen curd and storage time, and these samples could be discriminated easily on the Trp-F axis. The main decrease in Trp-F and so the main protein denaturation was induced by FC 50% at T9, while the highest Trp-F levels characterized fresh Mozzarella cheese without frozen curd (T1 0%). Samples containing an increasing percentage of frozen curd were characterized by higher FAST index values related to a decrease in Trp content, while Mozzarella cheese not containing frozen curd had the lowest FAST index both at T1 and T9 storage times.

A positive correlation was found between Trp-F and antioxidant activity (r = 0.67; *p* < 0.0001), while an inverse correlation was found between Trp-F and redox potential (r = −0.86; *p* < 0.0001), showing that the Trp-F level was higher when the antioxidant activity was higher and the redox potential was lower. An inverse correlation was found both between the FAST index and antioxidant activity (r = −0.73; *p* < 0.0001) and between Trp-F and carbonyl content (r = −0.78; *p* < 0.0001), suggesting that Trp-F and FAST index determination is a useful tool for evaluating the antioxidant and protein oxidation levels in Mozzarella cheese samples. To determine these two indicators, the method used requires quick and easy sample preparation and a fluorescence spectrophotometer reading, making it comparable to routine analysis.

#### 2.2.6. Discriminant Analysis

**Figure 4:**
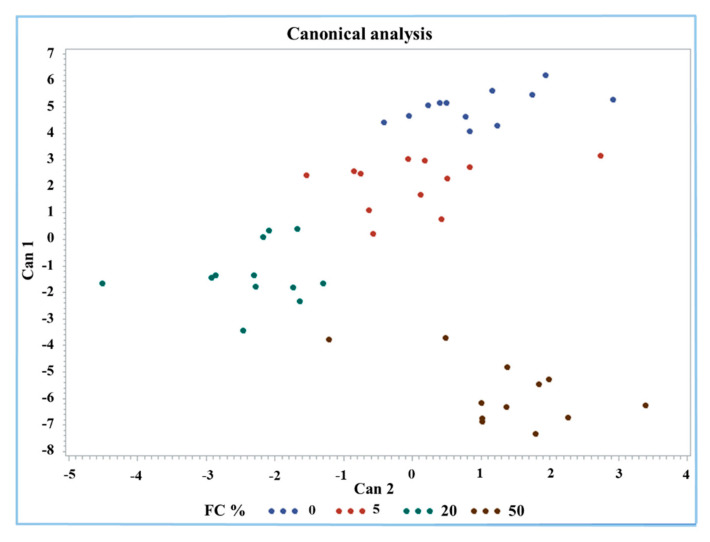
Canonical plot of discriminant analysis of buffalo Mozzarella cheese with respect to FC%.

A discriminant analysis was performed by the Candisc procedure for an overview and a classification of Mozzarella cheese according to FC%. All the 13 measured parameters were considered as variables. The discriminant analysis generated three canonical variables (Cans) where the first variable (Can1) explained 86.8% of the variance of the dataset. The first two Cans used for sample classification and visualization explained the largest amount of variance (97.7%), at 86.8% and 10.9%, respectively (Figure 4). The factors that mostly affect Can1 were protein, fat, antioxidant activity, Trp, FAST index, MDA, and carbonyls.

The group containing 50% FC was clearly identified by the analysis; in fact, it was classified separately in the lower right part of the graph (brown dots). Only one value was found near the part of the graph occupied by the group containing 20% FC (green dots). The groups with 0% and 20% FC were separated from each other but seemed to have a continuous downward trend for both Cans. However, the 0% and 50% frozen curd Mozzarella cheeses were clearly classified considering the first two Cans mainly due to protein, fat, Trp, carbonyls, and MDA content and the antioxidant activity and FAST index.

#### 2.2.7. Interactions between MDA and Carbonyl Content

The data shows that the high presence of frozen curd induced oxidation of both fat and protein. During the production of Mozzarella cheese, the use of frozen curd in high concentration negatively influenced the components that protect against oxidation. An inverse correlation was found among antioxidant activity and MDA content (r = −0.57; *p* < 0.0001), demonstrating the protection from lipid oxidation related to antioxidants content. Instead, a high positive correlation between redox potential and carbonyl content (r = 0.88; *p* < 0.0001), suggesting a relation between redox potential increase and susceptibility to protein oxidation both at different frozen curd percentages and during storage.

During storage for up to nine days, there was only protein oxidation while the fat was protected. Also, the results of Citta et al. [13] on the shelf life of yogurt showed a different behavior of lipid and protein oxidation; the addition of exogenous antioxidants had a protective effect only against lipid peroxidation. On the contrary, protein oxidation was not prevented and was associated with a decrease in antioxidant activity. However, after prolonged storage, the increase in antioxidant capacity was observed and attributed to the production of bioactive peptides.

There are both internal and external factors that induce susceptibility to oxidation. Among endogenous factors which affect oxidation are the composition of proteins and lipids, vitamin content, and antioxidant enzymes as catalase and glutathione peroxidase–reductase system [19]. Among external factors are heat treatment, cheese-making process, exposure to oxygen, storage length and conditions. The decrease in antioxidant components, i.e., vitamin losses during the processing and storage of foods, depends on the sensitivity of these composts to temperature, oxygen, light, pH, or combinations of these factors.

The major liposoluble constituents of milk with the function of protecting lipids from oxidation are tocopherols. Although tocopherols are sensitive to oxidation too, α-tocopherol, the most important component of vitamin E activity, is quite resistant to the effects of temperature and its protective effect persists during cheese storage [3,14]. It should also be added that little information exists on the possible effects of freezing on tocopherols degradation [28]. The α-tocopherol, resistant to temperature and soluble in the fat, therefore in direct contact with the fat, induces the protection of the fat from oxidation during product storage. It would be interesting to study the oxidation of fats during the entire shelf life of Mozzarella cheese, which in many cases can reach up to three weeks.

In any case, proteins are more exposed to denaturation and structural changes that make them more susceptible to oxidative phenomena due to external factors such as heat treatments or freezing. In particular, the formation of ice crystals during freezing causes coalescence due to the breakdown of fat globule membranes, while casein micelles lose their stability, inducing flocculation. The extent of these alterations varies according to the kind of freezing and thawing [61]. The interaction between the endogenous and exogenous factors that act on oxidation during freezing, as well as the correlation between the oxidation of proteins and lipids in dairy products, require further study.

## 3. Materials and Methods

### 3.1. Reagents and Standards

DPPH (1,1-diphenyl-2-picryl-hydrazyl radical), Trolox (6-hydroxy-2,5,7,8-tetramethylchromane-2-carboxylic acid), BHT (butylhydroxytoluene), TBA (2-thiobarbituric acid), TMP (1,1,3,3-tetra-methoxy-propane), DNPH (di-nitrophenol hydrazine), and BSA (bovine serum albumin) were purchased from Sigma-Aldrich (Steinheim, Germany).

Guanidine hydrochloride, TCA (trichloroacetic acid), acetonitrile, ethanol, ethyl acetate were purchased from Carlo Erba Reagents (Val de Reuil Cedex, France).

### 3.2. Buffalo Mozzarella Cheese Production

**Figure 5:**
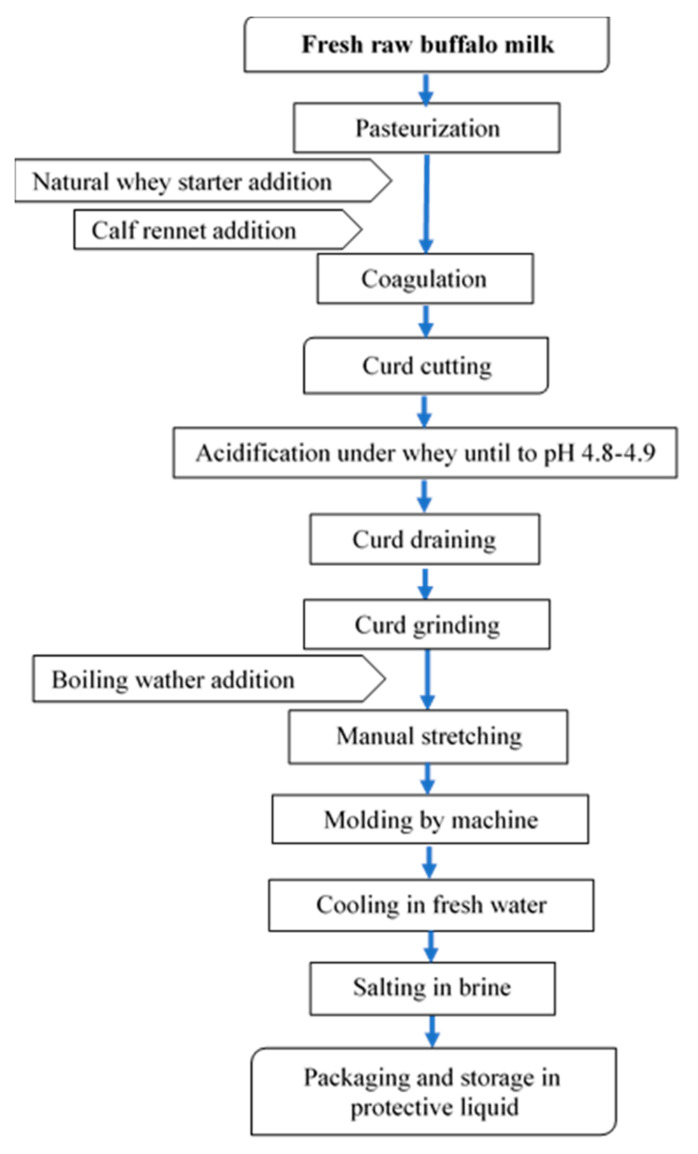
Flow-chart for buffalo Mozzarella cheese-making.

Buffalo Mozzarella cheese was produced in a medium-sized dairy plant located in the South of Italy. The process used in the dairy plant is reported in Figure 5.

The frozen curd (FC), used in the experimental trial, was produced in the same dairy plant. Fresh curd was frozen after the acidification, draining, and grinding phases, and packaged in a 4–5 kg block. The freezing phase was carried out in a freezing room at −20 °C and lasted about 3–4 h. The frozen curd was stored for about three months. The thawing phase occurred in tap water for 4 h. According to the experimental design, Mozzarella cheese samples were produced with different percentages of frozen curd (0%, 5%, 20%, and 50%) added to fresh curd for three repetitions. After being defrosted, the curd was added to fresh curd ready to be stretched, and they were stretched together and molded. At the end of the process, our samples were packaged in preserving liquid.

We used three trials, and each time 20 Mozzarella samples weighing 100 g each were collected for each group. During the trials, samples of frozen and fresh curd were also collected. Fresh Mozzarella cheese and curd samples were carried to the laboratory of the Council for Agricultural Research and Economics, Research Centre for Animal Production and Aquaculture of Monterotondo, Rome, Italy, for the analysis. Half of the samples were processed for analyses at one day of production (T1), and the other samples were stored at refrigeration temperature (4 °C) for nine days (T9).

### 3.3. Analyzed Samples

The analyses were carried out both on the fresh and stored product. The pH and redox potential were determinate on whole Mozzarella samples, and then the material was homogenized, divided into sub-samples, and frozen at −80 °C for chemical and oxidation analysis. Fresh and frozen curd samples from each repetition were treated in the same way. For each analysis, at least two repetitions of each sample were performed.

### 3.4. Physical and Chemical Analysis

Mozzarella cheese samples were submitted to the following analyses: pH, moisture [62] (IDF, 1986), ashes [63] (AOAC, 2000), protein, and fat [64] (ISO 21543:2006; IDF 201:2006).

### 3.5. Redox Potential Determination

For each sample, the redox potential was determined using a potentiometric method that uses a pH meter (Metrohm 827 pH Lab) equipped with a platinum electrode (combined Pt-ring 6.0451.100). The potential was expressed in millivolts (mV).

### 3.6. Antioxidant Activity—DPPH Method

The antioxidant activity of the samples was evaluated by DPPH (1,1-diphenyl-2-picryl-hydrazyl radical) method, as reported by Unal [5] on milk and cheese and by Tripaldi and al. [35] on Ricotta cheese.

Mozzarella sample (2 g) was mixed with 8 mL of 0.11 mM DPPH ethanolic solution. As a control, 2 mL of ethanol, in place of the sample, were added to 8 mL of 0.11 mM DPPH solution. The mixtures were stirred vigorously, allowed to react at room temperature for 20 min in the dark, and centrifuged for 10 min at 9000 *g* at 22 °C. Determinations of absorbance at 517 nm were performed on the supernatant by a double beam UV-VIS spectrophotometer (Lambda 25, PerkinElmer).

The antioxidant activity was calculated using the following equation

% antioxidant activity = (A_0_ − As)/A_0_ × 100

where A_0_ is the absorbance of the control and As is the absorbance of the tested sample.

The absorbance decreases when the DPPH radical is reduced by the antioxidant molecules of the sample, and therefore, the antioxidant activity expresses the ability of the sample to inhibit the DPPH radical. Using Trolox as standard, the antioxidant activity of the samples was expressed in Trolox equivalents (mmol eq Trolox/100 g).

### 3.7. Malondialdehyde Analysis

Malondialdehyde (MDA) determination is based on the reaction whit TBA (2-thiobarbituric acid) and on the detection of MDA-TBA fluorescent complexes. MDA analysis was performed according to Raia et al. [16] with some modifications as reported by Tripaldi and al. [35].

Mozzarella cheese samples (0.4 g) were homogenized in 3.4 mL of 10% *w/v* trichloroacetic Acid (TCA), adding 0.20 mL of BHT (butylhydroxytoluene) 2.8% *w/v* ethanolic solution as antioxidant. The mixtures were heated at 90 °C for 30 min, cooled in an ice bath for 20 min, and centrifuged at 10,000× *g* for 10 min to precipitate proteins. Supernatant aliquots (300 μL) were added to 700 μL of 0.28% *w/v* TBA and incubated at 90 °C for 30 min to induce the formation of TBA-MDA complexes. After filtration through a 0.22 µm filter, the samples were analyzed by HPLC analysis.

HPLC analysis was performed by Shimadzu-SPD-M10A HPLC with fluorimeter (RF-10A) and ZORBAX Eclipse Plus C18 column (4.6 mm × 250 mm × 5 µm) using isocratic mobile phase consisting of 5 mM sodium phosphate buffer (pH 7.0) and acetonitrile (80:20 *v*:*v*). Aliquots of 20 μL were injected and analyzed using a flow rate of 1 mL/min at room temperature. The fluorescent detector was set at an excitation wavelength of 515 nm and emission at 543 nm.

The MDA standard was prepared by dissolving 10 μL of tetra-methoxy-propane (TMP) in 10 mL of 0.1N HCl, incubating into a boiling water bath for 5 min and quickly cooling in ice. An aliquot (1 mL) of the hydrolyzed solution was diluted to 100 mL with water, and the resulting MDA standard was further diluted to concentrations ranging from 100 to 20 μg/L. The concentration of MDA in samples was then calculated using a calibration curve, and the obtained values were expressed as nmol/g.

### 3.8. Carbonyl Determination

Carbonyl content was determined using the method reported by Fedele and Bergamo [3] and Tripaldi and al. [35]. The method is based on the derivatization of the carbonyl group, with the di-nitrophenol hydrazine (DNPH), and the produced chromophore can be detected at 360 nm.

Mozzarella cheese samples (2 g) were mixed to 5 mL of 0.2 M sodium citrate-NaOH (pH 8) and aliquots (0.1 mL) were incubated with 0.4 mL of 10 mM DNPH in 2.5 M HCl. As control, aliquots (0.1 mL) were added to 0.4 mL of 2.5 M HCl without DNPH (blank).

After incubation for 30 min, in the dark at room temperature, to each sample, 0.5 mL of cold 20% *w/v* trichloroacetic Acid (TCA) was added to cause protein precipitation, and after 20 min in ice, the mixture was centrifuged for 10 min at 10,000× *g* at 4 °C, and 400 μL of 10% TCA was added to the pellet. Then the pellet was washed two times with 1 mL of ethanol/ethyl acetate (1:1 *v*/*v*) solution by centrifugation at 10,000× *g* for 10 min to remove free DNPH, and the pellet containing protein precipitates was finally dissolved in 1 mL of 6 M Guanidine hydrochloride in 10 mM phosphate buffer (pH 2.3). After incubating for 30 min in a 60 °C water bath, the absorbance at 370 nm was measured using a double-beam UV-VIS spectrophotometer (Lambda 25, PerkinElmer).

The protein concentration was determined by measuring the absorbance at 280 nm on the control without DNPH, using bovine serum albumin (BSA) as a standard (0.5–2.5 mg/mL).

The concentration of protein-bound carbonyls was expressed as nmol/mg of proteins, considering the molar extinction coefficient of 22,000 M^−1^ cm^−1^, according to the following formula

nmol/mg protein = (ΔAbs 360/22 mM) × 1000/C prot

where ΔAbs 360 = Abs 360 (sample with DNPT) − Abs 360 (blank) and C prot is the protein content (mg/mL).

### 3.9. FAST Method

The FAST method (Fluorescence of Advanced Maillard products and Soluble Tryptophan) has been used in previous works to discriminate heat-treatments in milk [56,57]. However, it was found that the degree of denaturation of whey proteins increases during frozen storage [58].

Mozzarella cheese sample (0.5 g) was mixed to 4.5 mL sodium acetate buffer (0.1 M, pH 4.6) and shanked vigorously for 30 s. After centrifugation at 4000× *g* for 10 min at room temperature, the supernatant was diluted (1:10), filtered through a 0.45 µm filter, and analyzed by Spectrofluorometer (FP-6300, Jasco).

Two fluorescence intensities were measured:

- Fluorescence of tryptophan at 290 nm excitation and 340 nm emission (Trp-F)
- Fluorescence of advanced Maillard’s products at 350 nm excitation and of 440 nm emission (AMP-F) and expressed in arbitrary units (a.u)

The FAST index was calculated as follows:

(AMP-F/Trp-F) × 100

In acidic conditions (pH 4.6), caseins precipitate, and some whey proteins also precipitate if denatured by heat or freezing. Trp-F measurement is also an estimate of the content of soluble whey proteins present in the supernatant and therefore of the undenatured proteins.

Samples containing more denatured protein are characterized by lower Trp-F values and higher FAST indexes. When advanced Maillard’s reaction occurs, samples present higher AMP-F values and higher FAST indexes.

### 3.10. Statistical Analysis

All data were analysed using the following linear model: Yijk = μ + FCi + Tj + (FC × T)ij + eijk, where μ = general mean; FC = frozen curd; T = storage; e = residual error. Statistical analysis was performed using the General Linear Model (GLM) procedure in SAS software version 9.3 [65]. The level of significance was set at *p* < 0.05. We also analyzed all the data with a correlation procedure (Proc Corr by SAS software); the results were listed in the text. The redox potential and carbonyls content were showed by Boxplot procedure (SAS software) on frozen curd by storage because the interactive effects were significant in the linear model. All data were analyzed with canonical correlation using the CANCORR procedure in SAS software version 9.3 [65].

## 4. Conclusions

The results show that the addition of frozen curd to fresh curd during the buffalo Mozzarella cheese production affected the chemical composition and oxidative stability of the product. Under the specific conditions of the experiment, when the percentage of frozen curd increased, the protein content increased, and the fat decreased. The reduced fat content could be due to both the lower fat content of the frozen curd and some factors of the freezing and thawing process of the curd, which may have affected the recovery of fat in the cheese. The percentage of fat in buffalo Mozzarella cheese is variable, and as a reference point, there are the PDO specifications where the minimum threshold of fat on the dry matter is 52%. All experimental samples were well above this fixed threshold.

The data also showed that the increased presence of frozen curd induces greater oxidation of fat and protein. On Mozzarella samples stored for nine days, the carbonyl level increased while the MDA was stable. In any case, the MDA content was always below the data of other dairy products and far below the threshold indicated by some authors. The carbonyl levels were similar to those found in some cooked cheeses.

The greater protection of fat against oxidative phenomena during product storage is confirmed by the negative correlation between MDA values and antioxidant activity. Therefore, in our samples of Buffalo Mozzarella, the high-fat content did not appear to negatively affect the antioxidant activity during storage limited to nine days.

The greater susceptibility of the protein to oxidative phenomena is probably due to the greater exposure of this component to denaturation and structural changes, and the predisposing factors included freezing and refrigerated storage.

Trp content results were sensitive to frozen curd addition and were comparable to routine analysis, which could be a valid indicator of the presence of frozen curd, even if further checks are necessary.

Understanding the mechanisms of lipid and protein oxidation during frozen curd storage could clarify the relevance of such reactions on product quality and the further development of antioxidant strategies to control oxidative damage in Mozzarella cheese processing and contrast its potential negative impact on cheese quality.

## Figures and Tables

**Table 1:** Chemical characteristics of fresh and frozen curd.

Parameter	pH	Moisture %	Ashes %	Protein g/100 g	Fat g/100 g	Fat/Protein
Fresh curd	4.97 b	43.4 b	1.83 a	16.2	30.07 a	1.88
Frozen curd	5.13 a	46.6 a	1.47 b	15.9	29.10 b	1.86
*p*-values	<0.0001	<0.005	<0.0001	n.s.	<0.05	n.s.

Data are expressed as the mean of three repetitions. For each parameter, means followed by a different letter in the row are significantly different. n.s. = not significant.

**Table 2:** Chemical characteristics of buffalo Mozzarella cheese produced with frozen curd (FC) preserved for one (T1) and nine days (T9).

FC%	pH		Moisture %		Ashes %		Protein g/100 g		Fat g/100 g		Fat/Protein	
0	5.39		64.0	a	1.73		13.9	b	22.6	a	1.63	a
5	5.39		64.1	a	1.73		14.3	ab	22.4	a	1.56	a
20	5.38		63.2	b	1.78		14.5	ab	22.1	a	1.52	ab
50	5.42		63.8	ab	1.80		15.1	a	20.9	b	1.39	b
Storage												
T1	5.34	b	60.8	b	1.65	b	15.4	a	23.7	a	1.54	
T9	5.44	a	66.8	a	1.87	a	13.5	b	20.4	b	1.52	
Significance	***p*-values**											
FC%	n.s.		< 0.05		n.s.		<0.0001		<0.01		<0.0001	
Storage	<0.0001		<0.0001		<0.05		<0.0001		<0.0001		n.s.	
Interaction FC% × Storage	n.s.		n.s.		n.s.		n.s.		n.s.		n.s.	

Data are expressed as mean (*n* = 6) for FC% and as mean (*n* = 12) for storage time. Means followed by a different letter in the column are significantly different. n.s. = not significant.

**Table 3:** Oxidative characteristics of fresh and frozen curd.

Parameter	Redox Potential mV	Antioxidant Activity mmol eq Trolox/100 g	MDA nmol/g	Carbonyls nmol/mg Protein	Trp-F a.u.	AMP-F a.u.	FAST Index
Fresh curd	163 b	10.3 a	0.67 b	1.54 b	420 a	37	8.0 b
Frozen curd	226 a	9.4 b	1.22 a	2.13 a	370 b	40	10.7 a
*p*-values	<0.0001	<0.05	<0.0001	<0.01	<0.001	n.s.	<0.0001

Data are expressed as mean of three repetitions. For each parameter, means followed by a different letter in the row are significantly different. n.s. = not significant; Trp-F = tryptophan fluorescence, AMP-F = Maillard’s Advanced Products fluorescence; FAST = Fluorescence of Advanced Maillard products and Soluble Tryptophan.

**Table 4:** Oxidative characteristics of buffalo Mozzarella cheese produced with frozen curd (FC) preserved for one (T1) and nine days (T9).

FC%	Redox Potential mV		Antioxidant Activity mmol eq Trolox/100 g		MDA nmol/g		Carbonyls nmol/mg Protein		Trp-F a.u.		AMP-F a.u.		FAST Index	
0	186	b	10.08	A	0.36	c	2.10	bc	385	a	56.3		14.7	b
5	192	ab	9.50	Ab	0.49	b	2.29	b	372	ab	57.8		15.6	ab
20	199	a	9.21	Ab	0.58	b	2.82	a	345	bc	59.3		17.2	ab
50	210	a	8.81	B	0.85	a	3.12	a	334	c	60.5		18.2	a
Storage														
T1	179	b	9.53		0.55		2.00	a	385	a	62.5	a	16.3	
T9	215	a	9.26		0.59		3.16	b	334	b	54.5	b	16.5	
Significance	***p*-values**													
FC%	<0.0001		<0.0001		<0.0001		<0.0001		<0.0001		n.s.		<0.001	
Storage	<0.0001		n.s.		n.s.		<0.0001		<0.0001		<0.0001		n.s.	
Interaction FC% × Storage	<0.05		n.s.		n.s.		<0.01		n.s.		n.s.		n.s.	

Data are expressed as mean (*n* = 6) for FC% and as mean (*n* = 12) for storage time. Means followed by a different letter in the column are significantly different. n.s. = not significant.

## Data Availability

The data presented in this study are available on request from the corresponding author.

## Sample Availability

Samples of the compounds are not available from the authors.
